# The impact of sleep habits on emotional and behavioral problems, quality of life, caregiver depression, and burnout in children with autism spectrum disorder

**DOI:** 10.3389/fpsyt.2026.1918823

**Published:** 2026-08-05

**Authors:** Asiye Arici Gürbüz, Semiha Cömertoğlu Arslan, Seda Bozduman Çelebi, Hülya Binokay, Hatice Altun

**Affiliations:** 1 Department of Child and Adolescent Psychiatry, Dr. Ekrem Tok Mental Health and Diseases Hospital, Adana, Türkiye; 2 Department of Child and Adolescent Psychiatry, Faculty of Medicine, Kahramanmaras Sutcu Imam University, Kahramanmaras, Türkiye; 3 Department of Child and Adolescent Psychiatry, Başakşehir Çam and Sakura City Hospital, Istanbul, Türkiye; 4 Department of Biostatistics, Çukurova University School of Medicine, Adana, Türkiye

**Keywords:** autism spectrum disorder, behavioral problems, child health quality, maternal burnout, maternal depression, sleep disturbances

## Abstract

**Background:**

Sleep disturbances affect 40–80% of children with autism spectrum disorder (ASD), yet their concurrent associations with neurobehavioral functioning, health-related quality of life (HRQoL), and primary caregiver mental health remain insufficiently characterized within an integrated multivariate framework that includes typically developing controls. This limits clinicians’ ability to position sleep disturbance within the broader family burden associated with ASD.

**Methods:**

This cross-sectional case-control study included 101 children aged 6–12 years with DSM-5-confirmed ASD and 81 typically developing controls, together with their primary caregiving mothers. Sleep was assessed using the Children’s Sleep Habits Questionnaire (CSHQ), child behavior using the Strengths and Difficulties Questionnaire (SDQ), HRQoL using the KINDL, and maternal psychiatric morbidity using the Beck Depression Inventory (BDI) and Maslach Burnout Inventory (MBI). Multiple linear regression and bootstrap mediation analyses were performed.

**Results:**

Despite significantly longer total daily sleep duration, children with ASD had poorer scores across most CSHQ subscales, including bedtime resistance, night wakings, and parasomnias (*p* < 0.05), whereas daytime sleepiness was not significantly different (*p* = 0.075). Maternal depression and burnout were substantially higher in the ASD group (*p* < 0.001). In multivariable analysis, ASD diagnosis (*B* = −6.124, p = 0.001), SDQ total difficulties (*B* = −0.533, *p* < 0.001), sleep disturbance severity (*B* = −0.523, *p* < 0.001), and maternal burnout (*B* = −0.146, *p* = 0.016) independently predicted lower HRQoL. Sleep quality significantly mediated the associations of child behavioral difficulties (23.1%, *p* = 0.016), maternal depression (51.4%, *p* = 0.002), and maternal burnout (36.6%, *p* = 0.027) with HRQoL.

**Conclusion:**

Sleep disturbances in children with ASD were associated with greater behavioral difficulties, lower HRQoL, and poorer maternal mental health. Sleep quality also mediated the associations between child behavioral difficulties, maternal psychological well-being, and HRQoL. These findings highlight the importance of assessing sleep as part of comprehensive, family-centered care for children with ASD.

## Introduction

Autism spectrum disorder (ASD) is a common neurodevelopmental condition characterized by persistent deficits in social communication and reciprocal social interaction, together with restricted, repetitive patterns of behavior and interests (1, 2). However, the clinical phenotype of ASD extends well beyond its social-communicative core. Children on the spectrum frequently exhibit substantial disturbances in sleep architecture and circadian rhythmicity, and these disturbances are now recognized as among the most clinically burdensome features of the disorder (2). Although the prevalence of sleep problems among typically developing children ranges from 25% to 40%, estimates in children with ASD reach as high as 80% (3). Compared with their neurotypical peers, children with ASD show markedly higher rates of prolonged sleep-onset latency, bedtime resistance, and night awakenings, and the resulting circadian dysregulation often follows a chronic course rather than resolving with development (4, 5).

These disruptions in sleep architecture appear to be closely associated with adverse daytime clinical outcomes and reduced adaptive functioning. Insufficient and fragmented sleep may weaken neurocognitive regulatory capacity and has been shown to exacerbate externalizing symptoms, including hyperactivity, irritability, impulsivity, and aggression (6). Frequent night awakenings and shortened sleep duration, in particular, have been associated with meaningful increases in aggressive and stereotyped behaviors in this population (1, 3). Beyond this escalating cycle of behavioral deterioration, poor-quality sleep is associated not only with behavioral difficulties but also with impairments in pragmatic and structural language skills (7). Chronic sleep loss may therefore contribute to a substantial reduction in the overall quality of life of children with ASD relative to their typically developing peers.

The clinical implications of sleep disturbance are not confined to the child. Rather, they contribute to a broader systemic burden that affects primary caregivers and the family unit as a whole. Daytime care for a child with ASD already requires considerable parental effort, and the addition of nocturnal sleep disruption, hypervigilance, and repeatedly interrupted sleep cycles imposes a cumulative burden on parents (8). Maladaptive pre-bedtime resistance behaviors and recurrent night awakenings are particularly closely linked with maternal psychological distress and heightened parenting anxiety (9) Interrupted nocturnal sleep, combined with sustained vigilance, may directly affect the physical and mental health of mothers and, consequently, overall parental quality of life (Liu et al., 2021). When chronic sleep fragmentation occurs alongside limited socioeconomic resources and restricted access to support networks, it co-occurs with elevated clinical depressive symptomatology and contributes to the emergence of parental burnout syndrome (10).

The existing literature reveals an important unresolved gap. Studies addressing sleep difficulties in children with ASD, their behavioral problems, and the depression or burnout experienced by their families have largely examined these domains in isolation, through unidirectional frameworks, or within narrowly defined dyadic relationships (3). Available studies have typically been restricted to a single diagnostic category, most often ASD alone, and have relied on relatively modest sample sizes (2). Research remains limited regarding the concurrent and integrated evaluation of child quality of life, emotional-behavioral functioning, and primary caregiver depression and burnout, with sleep disturbance serving as the central explanatory axis and with comparison against a sufficiently large, well-characterized healthy control sample. The present study was designed to address this gap by comparing the sleep habits of children with ASD aged 6 to 12 years with those of healthy controls and examining the multidimensional implications of sleep disturbances for child emotional and behavioral functioning, child quality of life, and maternal psychiatric morbidity, including depression and burnout. In line with this aim, the following hypotheses were tested:

1. The prevalence and severity of sleep disturbances, particularly night awakenings and difficulties with sleep onset, will be significantly higher in children with ASD than in healthy controls.
2. In the ASD group, poorer sleep quality will be associated with increased emotional and behavioral difficulties in daytime functioning, such as hyperactivity and irritability, and with lower overall child quality of life.
3. The severity of child sleep problems will be positively associated with clinical depressive symptoms and burnout levels in primary caregivers and negatively associated with child quality of life.

## Methods

### Study design and setting

This cross-sectional case-control study was conducted from March 2024 to March 2025 at the Child and Adolescent Psychiatry Outpatient Clinic of Adana Dr. Ekrem Tok Mental Health and Diseases Hospital, a tertiary public mental health institution serving a large metropolitan region in southern Türkiye.

### Participants

The study included 182 children aged 6–12 years and their primary caregivers, all of whom were biological mothers. The patient group comprised 101 children diagnosed with ASD, and the control group comprised 81 age-matched healthy children.

All ASD diagnoses were established by child and adolescent psychiatrists according to DSM-5 criteria, based on structured clinical evaluation and detailed developmental history assessment. Inclusion criteria for the ASD group were age between 6 and 12 years, a confirmed diagnosis of ASD, and completion of all study procedures. Children in the control group were required to have no current or lifetime history of psychiatric, neurological, metabolic, or genetic disorders. Participants with more than 10% missing data in the primary assessment measures were excluded from the analyses.

All study data were obtained from the children’s primary caregivers, who were their biological mothers. Mothers with physical, cognitive, or psychiatric conditions that could interfere with participation or comprehension of the assessment tools were excluded.

### Procedure

Data collection was completed during a single outpatient visit. All questionnaires were completed by the mothers; in cases of literacy difficulties, the researcher read the items aloud and recorded the responses verbatim. All forms were reviewed immediately after completion, and incomplete protocols were excluded from the final analyses.

Written informed consent was obtained from all parents or legal guardians before participation. Written assent was also obtained from children in the control group.

### Measures

#### Sociodemographic data and clinical information form

A semi-structured form developed by the researchers was used to collect information on the children’s age, sex, educational status, speech and language abilities, psychiatric and medical history, and current medication use, as well as the families’ socioeconomic characteristics. Information regarding caregiver educational level and family structure was also recorded.

#### Children’s sleep habits questionnaire

CSHQ is a 33-item parent-report measure developed by Owens, Spirito, and McGuinn (2000) to assess sleep quality and patterns in children aged 4–12 years. The scale comprises eight subscales: Bedtime Resistance, Sleep Onset Delay, Sleep Duration, Sleep Anxiety, Night Wakings, Parasomnias, Sleep-Disordered Breathing, and Daytime Sleepiness, as well as a total sleep disturbance score. A total score of > 41 suggests sleep disturbance. The Turkish version was validated by Fiş et al., 2010 (11) with acceptable internal consistency (α = 0.73).

#### Strengths and difficulties questionnaire — parent form

Twenty-five items distributed across five subscales; emotional symptoms, conduct problems, hyperactivity/inattention, peer problems, and prosocial behaviour. Higher scores on the first four subscales reflect greater difficulty; the prosocial subscale is scored inversely. Turkish reliability and validity were established by Güvenir et al., 2008 (12).

#### Children’s quality of life questionnaire (KINDL)

Six domains (physical well-being, emotional well-being, self-esteem, family, friends, school) are each assessed by four items, with scores transformed to a 0–100 scale; higher values indicate better health-related quality of life. KINDL used for the assessment of healthy children and children with a chronic disease, both in clinical and non-clinical settings The Turkish adaptation was performed by Eser et al., 2008 (13).

#### Maslach burnout inventory

Caregiver burnout was evaluated utilizing the MBI. Although the original instrument conceptualizes burnout across three classical subscales (emotional exhaustion, depersonalization, and personal accomplishment), the structural framework was modified in accordance with the psychometric properties of Turkish parental populations. The initial Turkish adaptation of the scale was established by Ergin et al., 1992 (14), and its applicability to primary caregivers of children with developmental disabilities was subsequently validated by Duygun and Sezgin 2003 (15). Crucially, structural factor analyses conducted by Duygun and Sezgin 2003 (15) demonstrated that a two-factor model provides a significantly superior statistical fit for parental cohorts, explaining 35.6% of the total variance.

In addition to the two subscales, the MBI Total Score was calculated by aggregating these dimensions and utilized across the statistical models to represent the cumulative severity of parental burnout, in line with established clinical literature.

#### Beck depression inventory

This 21-item self-report scale measures depressive symptom severity in primary caregivers, with total scores ranging from 0 to 63. Turkish adaptation and normative data were established by Hisli et al., 1988 (16).

#### Autism behavior checklist

The ABC is a 57-item instrument developed to evaluate the severity and frequency of autism-related behaviors. It consists of five subscales: sensory, relational, body and object use, language, and social self-care skills. Higher scores indicated more severe symptoms. The Turkish adaptation demonstrated strong reliability (17).

### Ethical considerations

Written informed consent was obtained from the mothers prior to participation. In addition, assent was sought from child participants when appropriate, in accordance with ethical guidelines. Participation was voluntary; caregivers were informed that withdrawal at any stage would have no bearing on the child’s clinical care. The study was conducted in accordance with the principles outlined in the Helsinki Declaration, and ethical approval was granted by the Adana City Hospital Training and Research Hospital Scientific Research Ethics Committee (Approval no Blinded statement).

### Statistical analysis

Categorical variables were summarised as frequencies and percentages, while numerical variables were expressed as median (Q1, Q3). Between-group comparisons of categorical variables were performed using the Pearson chi-square test. The assumption of normality was evaluated using the Kolmogorov–Smirnov test together with skewness and kurtosis values. Given the non-normal distribution of the numerical data, between-group comparisons of these variables were conducted using the Mann–Whitney U test, and associations among numerical variables were examined using Spearman’s rank-order correlation coefficient.

A multivariable linear regression model was constructed to identify the clinical and sociodemographic determinants of child quality of life, with age, sex, ASD diagnosis, SDQ total score, sleep quality (CSHQ total score), KINDL total score, maternal BDI total score, MBI total score, and socioeconomic status entered as predictors. In addition, mediation analyses were conducted to examine whether sleep quality served as a mediating variable in the associations of SDQ scores, maternal depression, and maternal burnout with child quality of life. Mediation modelling was performed using the *medmod* module in Jamovi version 2.4.6, and all other statistical analyses were carried out using IBM SPSS Statistics, version 20.0. The threshold for statistical significance was set at *p* < 0.05 for all analyses.

## Results

The sociodemographic, clinical, and family-related characteristics of the patient and control groups are compared in **Table 1**. The ASD group showed considerable clinical heterogeneity. Overall, 73.3% of participants had co-occurring intellectual disability, including 49.5% with mild and 23.8% with moderate intellectual disability. Regarding language abilities, 44.6% of participants were able to communicate using sentences, whereas 40.6% used single words and 14.9% had no meaningful speech. In addition, 65.3% had at least one comorbid psychiatric disorder, and approximately 60% were receiving antipsychotic-containing treatment. The groups did not differ significantly in sex distribution, comorbid enuresis, comorbid physical illness, maternal educational attainment, maternal employment status, specific subtypes of maternal and paternal psychiatric disorders, paternal employment status, family structure, or place of residence (*p* > 0.05). By contrast, paternal educational attainment was significantly lower in the patient group than in the control group (*p* = 0.001), and household economic level was also significantly lower in the patient group (*p* = 0.006). No statistically significant between-group differences were observed in parental age, child age, or bedtime (*p* > 0.05). However, median daily total sleep duration was significantly longer among children in the patient group than among those in the control group (*p* < 0.001).

**Table 1:** Comparison of sociodemographic, clinical, and family characteristics between the patient and control groups.

Category	Category	ASD n (%)	Control n (%)	p
**Sex**	Male	65 (64.4)	44 (54.3)	0.170
	Female	36 (35.6)	37 (45.7)	
**Educational status**	Special education only	24 (23.8)	0 (0.0)	<0.001**
	Receives both special education and formal education	68 (67.3)	0 (0.0)	
	Formal education only	9 (8.9)	81 (100.0)	
**Mild Intellectual Disability**	Absent	51 (50.5)	81 (100.0)	<0.001**
	Present	50 (49.5)	0 (0.0)	
**Moderate Intellectual Disability**	Absent	77 (76.2)	81 (100.0)	<0.001**
	Present	24 (23.8)	0 (0.0)	
**Co-occurrence of ASD and Intellectual Disability**	Absent	27 (26.7)	81 (100.0)	<0.001**
	Present	74 (73.3)	0 (0.0)	
**Speech status**	No speech or meaningless speech	15 (14.9)	0 (0.0)	<0.001**
	Has single words	41 (40.6)	0 (0.0)	
	Has sentences	45 (44.6)	81 (100.0)	
**Comorbid psychiatric disorder**	Absent	35 (34.7)	78 (96.3)	<0.001**
	Present	66 (65.3)	3 (3.7)	
**Type of comorbid psychiatric disorder**	Absent	39 (38.6)	78 (96.3)	<0.001**
	ADHD	27 (26.7)	2 (2.5)	
	Conduct disorder	18 (17.8)	1 (1.2)	
	Communication Disorder	17 (16.8)	0 (0.0)	
**Current medications**	No medication	38 (37.6)	81 (100.0)	<0.001**
	Antipsychotic	40 (39.6)	0 (0.0)	
	Psychostimulant	3 (3.0)	0 (0.0)	
	Antipsychotic + methylphenidate	17 (16.8)	0 (0.0)	
	Antipsychotic + atomoxetine	3 (3.0)	0 (0.0)	

*p<0.05,**p<0.001,*** p=(0.001). p value in bold is statistically significant.

A statistically significant difference was found between the patient and control groups in terms of medication use (p<0.001). In the patient group, 62.4% of the participants were using medication, whereas none of the participants in the control group were using medication. The proportion of participants not using medication was 37.6% in the patient group and 100.0% in the control group.

Between-group comparisons of SDQ, CSHQ, KINDL, MBI Emotional Exhaustion, MBI Personal Accomplishment score and BDI scores are presented in **Table 2**.

**Table 2:** Comparison of scale scores between the patient and control groups.

SDQ	ASD Median (Q1–Q3)	Control Median (Q1–Q3)	p
Emotional Symptoms	3 (2–5)	1 (0–3)	<0.001**
Conduct Problems	2 (1–4)	1 (0–1)	<0.001**
Hyperactivity/Inattention	6 (4–8)	3 (2–5)	<0.001**
Peer Problems	5 (3–6)	2 (2–4)	<0.001**
Prosocial Behavior	5 (3–7)	9 (7–10)	<0.001**
Total SDQ score	17 (13–21)	8 (5–11)	<0.001**
CSHQ			
Bedtime Resistance	10.00 (8.00–12.00)	8.00 (7.00–10.00)	<0.001**
Sleep Onset Delay	1.00 (1.00–2.00)	1.00 (1.00–1.00)	<0.001**
Sleep Duration	5.00 (4.00–6.00)	3.00 (3.00–5.00)	<0.001**
Sleep Anxiety	7.00 (5.00–8.00)	5.00 (4.00–7.00)	0.005*
Night Wakings	5.00 (4.00–6.00)	4.00 (3.00–5.00)	0.016*
Parasomnias	11.00 (9.00–12.00)	9.00 (7.00–10.00)	<0.001**
Sleep-Disordered Breathing	4.00 (3.00–5.00)	3.00 (3.00–4.00)	<0.001**
Daytime Sleepiness	13.00 (10.00–15.00)	12.00 (10.00–13.00)	0.075
Total sleep disturbance score	52.00 (45.00–59.00)	45.00 (40.00–51.00)	<0.001**
KINDL			
Physical well-being	15,00 (13,00–17,00)	16,00 (14,00–19,00)	0.056
Emotional well-being	15,00 (12,00–17,00)	17,00 (15,00–19,00)	<0.001**
Self-esteem	14,00 (12,00–15,00)	17,00 (15,00–18,00)	<0.001**
Family	15,00 (13,00–18,00)	18,00 (16,00–19,00)	<0.001**
Friends	12,00 (10,00–14,00)	16,00 (15,00–17,00)	<0.001**
School	14,00 (12,00-15,00)	17 (15,00-18,00)	<0,001^**^
Total KINDL score	80,00 (72,00–89,00)	99,00 (92,00–104,00)	<0.001**
MBI			
Emotional exhaustion	18,00 (8,00–24,00)	8,00 (5,00–14,00)	<0.001**
Personal accomplishment	8,00 (4,00–12,00)	*5,00 (3,00–8,00)*	*p=0.005*
Total score	24,00 (14,00–36,00)	13,00 (8,00–22,00)	<0.001**
BDI total score	14 (8–19)	4 (1–8)	<0.001**

Strengths and Difficulties Questionnaire (SDQ), Children’s Sleep Habits Questionnaire (CSHQ), Children’s Quality of Life Questionnaire (KINDL), Maslach Burnout Inventory (MBI),Beck Depression Inventory (BDI). *p<0.05,**p<0.001,*** p=(0.001). p value in bold is statistically significant.

The median Emotional Exhaustion score was significantly higher in the patient group than in the control group (18.00 [8.00–24.00] vs. 8.00 [5.00–14.00]; p<0.001). Similarly, the median reverse-scored Personal Accomplishment score, reflecting reduced personal accomplishment, was significantly higher in the patient group than in the control group (8.00 [4.00–12.00] vs. 5.00 [3.00–8.00]; p=0.005).

Regarding the ABC, the median total score in the patient group was 42.00 (16.00–79.00). The highest median subscale scores were observed in the *Relating and Social and Self-Help* domains [10.00 (2.00–18.00) and 10.00 (3.00–15.00), respectively]. Median scores for the remaining subscales were 9.00 (2.00–15.00) for *Language*, 7.00 (0.00–22.00) for *Body and Object Use*, and 5.00 (0.00–10.00) for *Sensory*.

Correlations among sleep parameters, health-related quality of life, parental burnout, and autistic symptom severity within the patient group are shown in **Table 3**. Correlation analyses showed that the CSHQ total score was significantly and positively correlated with the SDQ total difficulties score, MBI Emotional Exhaustion and Personal Accomplishment score, and ABC-based autistic symptom severity, and significantly negatively correlated with the KINDL quality-of-life score. Age was not significantly associated with any scale score.

**Table 3:** Correlation matrix between sleep (CSHQ), KINDL, burnout (MBI), and ASD-ABC scales within the patient group.

CSHQ total score	CSHQ total score	Age	SDQ total score	KINDL total score	MBI total score	ASD-ABC toplam	BDI total score
CSHQ total score	1	-0.185 (0.064)	0.442 (<0.001)**	-0.314 (0.001)**	0.506 (<0.001)**	0.227 (0.023)	0.341 (<0.001)**
Age		1	-0.032 (0.753)	0.042 (0.679)	-0.146 (0.145)	0.021 (0.832)	-0.185 (0.064)
SDQ total score			1	-0.439 (<0.001)**	0.326 (0.001)***	0.394 (<0.001)**	0.442 (<0.001)**
KIND Ltotal score				1	-0.286 (0.004)	-0.510 (<0.001)**	-0.341 (0.001)**
MBI total score					1	0.266 (0.007)	0.506 (<0.001)**
ASD-ABC total score						1	0.227 (0.023)
BDI total score							1

Strengths and Difficulties Questionnaire (SDQ), Children’s Sleep Habits Questionnaire (CSHQ), Children’s Quality of Life Questionnaire (KINDL), Maslach Burnout Inventory (MBI),Beck Depression Inventory (BDI), Autism Behavior Checklist (ABC) *p<0.05,**p<0.001,*** p=(0.001).

The multivariable linear regression analysis of clinical factors associated with child quality of life is presented in **Table 4**. In the final model, children with ASD had a mean KINDL score 6.124 points lower than that of children in the control group (*p* = 0.001). Higher SDQ total scores were associated with lower child quality-of-life scores. The CSHQ total score was also a significant negative predictor of child quality of life (*p* < 0.001); because higher CSHQ scores indicate poorer sleep quality, this finding suggests that greater sleep disturbance was associated with lower child quality of life. In addition, the MBI Emotional Exhaustion and Personal Accomplishment score were negatively associated with child quality of life (*p* = 0.016), indicating that higher caregiver/maternal burnout was associated with lower child quality-of-life scores.

**Table 4:** Multivariable model of clinical factors associated with KINDL.

Model	Model	B	SE	p
Step 1	Group ASD vs control	-5.851	1.846	0.002
Step 1	Gender	-1.235	1.495	0.410
Step 1	Age	-0.433	0.406	0.288
Step 1	Family socioeconomic status: (Category 2 vs. 1)	-1.284	2.475	0.604
Step 1	SDQ total score	-0.509	0.137	<0.001**
Step 1	Maternal BDI total score	-0.043	0.112	0.700
Step 1	Sleep quality total score	-0.524	0.093	<0.001**
Step 1	MBI total score	-0.149	0.067	0.028
Step 6	Group ASD vs control	-6.124	1.756	0.001***
Step 6	SDQ total score	-0.533	0.132	<0.001**
Step 6	Sleep quality total score	-0.523	0.089	<0.001**
Step 6	MBI total score	-0.146	0.060	0.016

Strengths and Difficulties Questionnaire (SDQ), Children’s Sleep Habits Questionnaire (CSHQ), Children’s Quality of Life Questionnaire (KINDL), Maslach Burnout Inventory (MBI),Beck Depression Inventory (BDI), Autism Behavior Checklist (ABC) *p<0.05,**p<0.001,*** p=(0.001).

The mediation analysis results are presented in **Table 5**.

**Table 5:** Mediation analyses examining sleep quality as a mediator of the associations between GGA, maternal depression, parental burnout, and child quality of life.

Independent Variable	Effect/Path	Path Label	Coefficient	SE	%95 CI	p	Mediation(%)
**SDQ total score**	**Indirect effect**	a × b	-0.215	0.090	-0.390 – -0.039	0.016	23.1
	**Direct effect**	c	-0.717	0.167	-1.043 – -0.390	<0.001**	76.9
	**Total effect**	c + a × b	-0.931	0.179	-1.282 – -0.581	<0.001**	100.0
	**SDQ total score → Sleep quality**	a	0.417	0.151	0.120 – 0.714	0.006	—
	**Sleep quality → Child quality of life**	b	-0.515	0.106	-0.722 – -0.308	<0.001**	—
**Maternal BDI total score**	**Indirect effect**	a × b	-0.226	0.073	-0.368 – -0.084	0.002	51.4
	**Direct effect**	c	-0.214	0.126	-0.460 – 0.032	0.089	48.6
	**Total effect**	c + a × b	-0.440	0.128	-0.691 – -0.188	<0.001**	100.0
	**Maternal BDI total score → Sleep quality**	a	0.405	0.098	0.213 – 0.596	<0.001**	—
	**Sleep quality → Child quality of life**	b	-0.559	0.118	-0.790 – -0.327	<0.001**	—
**MBI total score**	**Indirect effect**	a × b	-0.090	0.041	-0.170 – -0.010	0.027	36.6
	**Direct effect**	c	-0.156	0.074	-0.301 – -0.012	0.034	63.4
	**Total effect**	c + a × b	-0.247	0.081	-0.404 – -0.089	0.002	100.0
	**MBI total score → Sleep quality**	a	0.156	0.064	0.031 – 0.281	0.015	—
	**Sleep quality → Child quality of life**	b	-0.580	0.112	-0.798 – -0.361	<0.001**	—

SDQ, Strengths and Difficulties Questionnaire; BDI, Beck Depression Inventory; Sleep quality (assessed via CSHQ total score) child quality of life (assessed via KINDL total score) is modeled as the dependent/outcome variable SE (Standard Error); CI (Confidence Interval) *p<0.05, **p<0.001, *** p=(0.001). p value in bold is statistically significant.

The mediating role of sleep quality in the association between the SDQ total score and child quality of life was examined. The SDQ total score significantly and positively predicted the CSHQ total score (*a* = 0.417, SE = 0.151, 95% CI: 0.120–0.714, *p* = 0.006), whereas the CSHQ total score significantly and negatively predicted child quality of life (*b* = −0.515, SE = 0.106, 95% CI: −0.722 to −0.308, *p* < 0.001). Given the directionality of CSHQ scoring, in which higher scores indicate poorer sleep quality, these findings indicate that higher SDQ total scores were associated with poorer sleep quality, which was in turn associated with lower child quality of life.

The mediating role of sleep quality in the association between maternal BDI scores and child quality of life was also evaluated. The maternal BDI total score was a significant positive predictor of the CSHQ total score (*a* = 0.405, SE = 0.098, 95% CI: 0.213–0.596, *p* < 0.001). Because higher CSHQ scores indicate poorer sleep quality, this finding suggests that higher maternal depressive symptom scores were associated with poorer sleep quality in the children.

The mediating role of sleep quality in the association between maternal MBI Emotional Exhaustion and Personal Accomplishment scores and child quality of life was likewise assessed. The MBI Emotional Exhaustion and Personal Accomplishment scores was a significant positive predictor of the CSHQ total score (*a* = 0.156, SE = 0.064, 95% CI: 0.031–0.281, *p* = 0.015). Consistent with the directionality of CSHQ scoring, this finding indicates that higher levels of caregiver/maternal burnout were associated with poorer sleep quality in the children.

**Figure 1:**
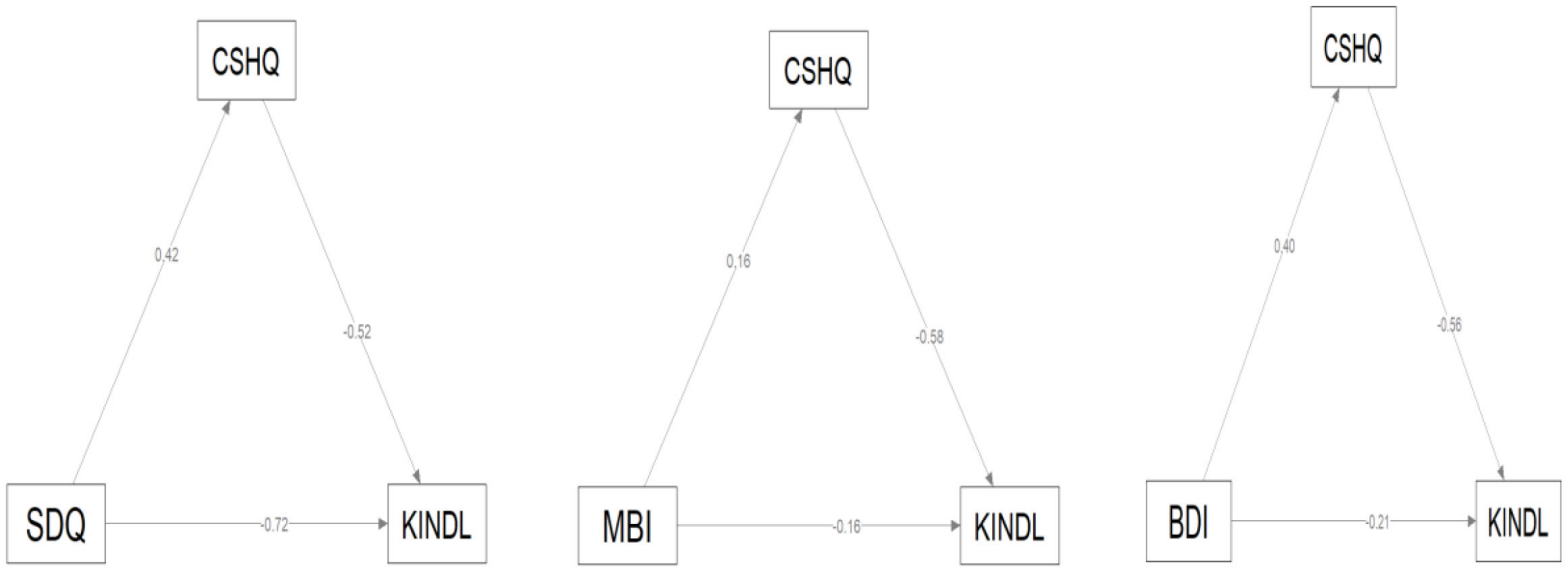
Statistical path diagrams illustrating the mediating role of sleep quality (CSHQ) in associations with child quality of life (KINDL).

Statistical path diagrams illustrating the mediating role of sleep quality (CSHQ) in associations with child quality of life (KINDL) are presented in **Figure 1**.

## Discussion

This study examined the interrelationships among sleep disturbances, behavioral functioning, health-related quality of life, and parental mental health in children diagnosed with ASD. Children with ASD exhibited substantially greater sleep difficulties than healthy controls. Notably, despite having a comparatively longer total sleep duration, the ASD group showed clinically meaningful disturbances in domains reflecting sleep quality rather than sleep quantity, including bedtime resistance, night awakenings, and parasomnias. The co-occurrence of sleep disturbance with elevated behavioral difficulties in the children and higher depressive symptoms and burnout in their caregivers suggests that sleep problems in ASD should be conceptualized not as an isolated nocturnal symptom cluster but as part of a multidimensional clinical burden embedded within family functioning.

### The sleep duration paradox

One of the most clinically notable findings of the present analysis was that children with ASD had a significantly longer daily total sleep duration than their typically developing peers, yet showed considerably poorer scores across all quality-oriented CSHQ subscales, including bedtime resistance, night awakenings, and parasomnias. This dissociation indicates that sleep duration alone provides an incomplete and potentially misleading clinical picture and highlights the possible contribution of pharmacological factors. A plausible explanatory pathway involves the high rate of antipsychotic exposure within the ASD group. Second-generation antipsychotic agents, which are frequently prescribed to manage disruptive behaviors and affective outbursts in this population, exert potent antagonism at histaminergic H_1_ and serotonergic 5-HT_2_A receptors and are well recognized for producing marked sedation and pharmacologically mediated hypersomnia (2, 18, 19). This medication-related effect may artificially extend parent-observed sleep duration while simultaneously disrupting sleep microarchitecture, suppressing REM activity, and attenuating slow-wave components (20). One possible explanation for the prolonged sleep observed in the patient group is antipsychotic medication exposure. In our cohort, approximately 60% of children with ASD were receiving antipsychotic-containing treatment. Previous studies have reported that antipsychotic exposure has been associated with diminished sleep quality in children (2). However, although medication use was recorded, medication dosage, treatment duration, and the specific relationship between medication exposure and sleep outcomes were not analyzed in the present study. Therefore, antipsychotic exposure should be considered only as a possible contributing factor to the prolonged sleep duration observed rather than a definitive explanation. Further studies incorporating detailed pharmacological data are needed to clarify this relationship. This pattern is more plausibly characterized as fragmented, pharmacologically influenced hypersomnia punctuated by frequent awakenings. Our findings align closely with contemporary sleep medicine literature, which emphasizes that the architectural integrity and continuity of sleep, rather than absolute duration alone, are more decisive determinants of daytime functioning (3). Parental reports of unusually long sleep duration in children with ASD therefore should not be interpreted as a reassuring health indicator. Instead, this pattern may serve as a clinical marker reflecting autism symptom severity, underlying psychiatric burden, and cumulative pharmacological exposure (3, 18). The clinical implication is that pediatric psychiatrists evaluating sleep in children with ASD should shift the assessment focus from how long a child sleeps to how well and how coherently sleep is maintained (3, 6).

### Sleep problems, behavioral difficulties, and quality of life

Our findings indicate that sleep disturbances in children with ASD are not confined to nocturnal hours but are closely associated with a neurobehavioral profile that compromises daytime functioning across multiple domains. Within the ASD group, greater sleep disturbance severity co-occurred with higher levels of emotional symptoms, conduct problems, hyperactivity, and peer relationship difficulties, as captured by the SDQ, alongside relatively lower prosocial behavior. Neurodevelopmental research has shown that insufficient and fragmented sleep is associated with disruptions in prefrontal inhibitory control, working memory, and amygdala-mediated emotional regulation (6). In ASD, in which the cortico-limbic regulatory networks underpinning these functions are already vulnerable, sleep fragmentation may function as an additional stressor on an already taxed neurodevelopmental system (18). The clinical correlate is amplified externalizing behavior, including heightened irritability, intensified stereotypy, weakened impulse control, and reactive aggression (1). This pattern is consistent with a substantial body of work identifying sleep disturbance as one of the most robust correlates of daytime behavioral difficulties in children with ASD (4, 6).

Beyond behavioral and emotional dysregulation, our findings extend this literature into the psychosocial domain. Sleep pathology was closely associated with meaningful reductions in the emotional, self-esteem, and relational dimensions of child quality of life as indexed by the KINDL. The strength and inverse direction of this association across the multidimensional KINDL framework, encompassing emotional well-being, relational functioning, self-esteem, and family domains, suggest that children with ASD who experience chronic sleep disruption may face cumulative impairments that extend beyond neurobehavioral symptomatology into core participation domains, including family life, peer interaction, and educational engagement (3, 7). Restorative and architecturally preserved sleep is increasingly understood as a foundational component of healthy psychosocial development in childhood. When this foundation is compromised, the clinical consequences may extend beyond incremental symptom burden and create a domain of chronic vulnerability affecting social participation, daily functioning, and broader quality of life (21).

### Caregiver burden: maternal burnout, depressive morbidity, and socioeconomic strain

The primary caregiving role for a child with ASD imposes a sustained and substantial psychosocial burden on the family system, even in the absence of sleep difficulties (22, 23). When daily life becomes organized around behavioral management, special education needs, and intensified caregiving demands, parental psychological resilience is challenged by chronic stress exposure rather than an acute stressor. Our sociodemographic findings suggest that this burden is not solely attributable to the child’s neurodevelopmental difficulties but is also intertwined with broader social disadvantage, including lower paternal educational attainment and constrained household economic resources in the patient group. Financial limitations may restrict access to professional services and respite care, leaving mothers more vulnerable both physically and psychologically and reinforcing an uneven distribution of caregiving responsibility within the household (24). Given the cross-sectional design, the directionality between these socioeconomic variables and maternal psychological burden cannot be determined, and both may plausibly reflect convergent expressions of a shared axis of disadvantage rather than a unidirectional relationship.

When child sleep pathology is superimposed on this baseline strain, the clinical burden becomes considerably more complex. Night awakenings and fragmented sleep patterns in the child are associated with chronic maternal sleep disruption, which has been linked to compromised emotional resilience, reduced stress tolerance, and altered autonomic regulation (25). In our data, mothers in the patient group exhibited notably higher levels of burnout and depressive symptomatology than mothers in the control group, suggesting that this strain may extend beyond subjective caregiving difficulty into a clinically meaningful domain of psychiatric burden. The heightened emotional exhaustion and diminished sense of personal accomplishment captured by the MBI point to systematic depletion of the psychological resources required to sustain the parenting role.

The strength of the association observed in our sample between sleep disturbance severity and maternal burnout and depressive symptoms suggests that these maternal presentations should not be regarded as independent comorbidities incidental to ASD. Rather, they may be better understood as clinical manifestations of an integrated family stress network shaped jointly by the child’s neurodevelopmental difficulties, heightened caregiving demands, and surrounding socioeconomic strain. The parallel patterning of sleep disturbance and behavioral dysregulation in our data is also consistent with a potentially bidirectional relationship between these domains. As behavioral dysregulation intensifies, daytime caregiving demands increase, while a depleted mother may be less able to provide the co-regulatory scaffolding her child requires, which may in turn further aggravate behavioral irritability and sleep difficulties in a self-perpetuating cycle (26, 27). However, the cross-sectional design of our study does not permit definitive inference regarding the directionality or causal sequencing of these relationships. With this caveat, early recognition of maternal depression and integration of caregiver-focused supportive interventions into the treatment plan represent reasonable strategies for protecting maternal mental health (21, 28). Such approaches may also constitute a meaningful component of family-centered interventions aimed at improving behavioral regulation and quality of life in children with ASD.

### The relational bridge between childhood sleep quality and daytime functioning

The multidimensional mediation analyses conducted in this study suggest that daytime emotional and behavioral difficulties in children with ASD are associated with lower health-related quality of life both directly and indirectly through nocturnal sleep quality. Across the mediation models, the indirect pathways through sleep quality reached statistical significance, supporting the interpretation that sleep disturbance may function as a meaningful relational bridge between daytime behavioral difficulties and reduced child quality of life. Our parent-reported observations are consistent with literature indicating that the emotional symptoms and externalizing behaviors frequently encountered in ASD are associated with diminished sleep quality and fragmented nocturnal architecture (3, 6). This dysfunctional sleep pattern, in turn, is plausibly associated with reduced adaptive and psychosocial functioning the following day, with cumulative repercussions for social participation and peer relationships (3, 18). Within the relational framework permitted by our cross-sectional data, poorer sleep quality emerges as a clinically relevant mediating component in the link between daytime behavioral difficulties and quality of life and warrants explicit attention in routine clinical follow-up.

### Caregiver mental health and parent–child dynamics

In the multivariable regression analyses examining maternal psychiatric morbidity, maternal burnout remained independently and negatively associated with child quality of life, even after accounting for clinical depressive symptoms, indicating that these two domains of psychosocial strain carry distinct clinical relevance (29). Interpreted within a cross-sectional framework, this pattern is consistent with the possibility that mothers experiencing clinically significant stress, low mood, or burnout may have greater difficulty sustaining the soothing and supportive parental co-regulation that children with ASD require during bedtime transitions and night awakenings (26, 27). The parental burden that emerges when this regulatory support cannot be reliably provided (30) is consistent with prior work indicating that child sleep difficulties are associated with sustained depressive symptoms and cumulative stress in caregivers (21, 31). Taken together, these findings suggest that maternal mental health difficulties and child sleep disturbances are unlikely to operate through a purely unidirectional pathway; rather, they appear to interact within a reciprocal network that shapes child quality of life.

### Limitations and future directions

Several methodological limitations should be considered. First, the cross-sectional design limits causal inference regarding the relationships among variables. Second, reliance on parent-reported measures introduces the possibility of shared method variance, which may have influenced the strength of the observed associations; longitudinal studies incorporating objective sleep assessments will therefore be essential to confirm these findings. Third, the absence of detailed information on the dosage and duration of psychopharmacological treatments received by the patient group limited more granular evaluation of the pharmacological contribution to prolonged sleep duration. Finally, recruitment from a single tertiary child psychiatry center may limit the generalizability of the findings to other sociocultural and clinical settings.

## Conclusion

These findings indicate that sleep disturbances in children with ASD are not merely nocturnally confined symptoms but multilayered clinical phenomena associated with child behavioral functioning, quality of life, and the mental health of the primary caregiver. Fragmented and poor-quality sleep in children with ASD was associated with higher levels of hyperactivity, irritability, and behavioral difficulties, and this pattern co-occurred with elevated burnout and depressive symptoms among caregiving mothers. Mothers should therefore be understood not as passive caregivers but as central therapeutic resources positioned at the intersection of behavioral regulation and family functioning. Screening for caregiver burnout and depressive symptoms could reasonably be incorporated into routine child and adolescent psychiatric assessment. Early recognition and comprehensive management of sleep difficulties in ASD therefore represent important clinical intervention points with implications for family functioning and the quality of life of children living with the condition.

## Data Availability

The raw data supporting the conclusions of this article will be made available by the authors, without undue reservation.
